# Automated pipeline for linear and volumetric assessment of facial swelling after third molar surgery

**DOI:** 10.1186/s12903-024-05193-7

**Published:** 2024-11-19

**Authors:** Selene Barone, Paolo Zaffino, Marianna Salviati, Michela Destito, Alessandro Antonelli, Francesco Bennardo, Lucia Cevidanes, Maria Francesca Spadea, Amerigo Giudice

**Affiliations:** 1 School of Dentistry, Department of Health Sciences, Magna Graecia University of Catanzaro, Viale Europa, Catanzaro, 88100 Italy; 2 Department of Experimental and Clinical Medicine, Magna Graecia University of Catanzaro, Viale Europa, Catanzaro, 88100 Italy; 3 School of Dentistry, Department of Orthodontics and Pediatric Dentistry, University of Michigan, Ann Arbor, USA; 4 Institute of Biomedical Engineering, Karlsruhe Institute of Technology (KIT), Karlsruhe, Germany

**Keywords:** 3D swelling analysis, Facial scanner, Third molar surgery edema, Volumetric measurements, Three-dimensional surface imaging

## Abstract

**Background:**

Extraction of mandibular third molars (M3Ms) is a routine procedure in oral and maxillofacial surgery, often associated with postoperative symptoms like pain, facial swelling, and trismus. This study aimed to introduce a standardized and automated protocol for swelling analysis following M3M surgery, presenting results regarding clinical conditions immediately and one-week after surgery.

**Methods:**

In a prospective study, 35 patients were enrolled (mean age: 24.4 ± 5.8 years) for removal of 54 M3Ms. Facial swelling was evaluated through 3D facial scans before surgery (T0), at three days (T1), and seven days (T2) post-surgery. The open-source software 3DSlicer facilitated automated analysis, including data anonymization, orientation, surface registration, qualitative comparisons, linear measurements, and volumetric quantification. Pairwise superimposition of facial models enabled qualitative, vectorial, and quantitative assessments, comparing initial conditions with swelling development at T1 and T2. Additionally, changes between T1 and T2 were also evaluated. Secondary outcomes encompassed clinical evaluations of pain, trismus (maximum mouth opening), and surgery time. Statistical analysis involved the paired Student t-test to assess longitudinal changes and analysis of variance to evaluate outcome variables concerning difficulty scores. Linear regression models correlated primary outcome variables with secondary study variables (α < 0.05).

**Results:**

Longitudinal analysis demonstrated significant but variable facial swelling, pain, and trismus at T1, followed by improvement at T2 (*p* < 0.001). Linear and volumetric differences correlated positively with surgery time (*p* < 0.05). A direct proportionality between linear and volume differences was observed, higher values at T1 correlated with higher values at T2 (*p* < 0.05).

**Conclusions:**

An innovative digital workflow for precise quantification of postoperative facial changes was implemented, incorporating volumetric measurements that surpass linear assessments. Clinical conditions demonstrated a direct correlation with surgery time, deteriorating immediately and improving one-week after surgery.

## Background

The removal of lower third molar (M3M) is one of the most frequent procedures in oral and maxillofacial surgery. It still represents a challenge for the surgeon because different variables can influence the choice of intervention, the approach for surgical procedure and the post-operative consequences. Quality-of-life immediately after surgery is mainly characterized by the occurrence of inflammatory processes with specific clinical signs and symptoms of pain, facial swelling, and trismus. Different factors can influence the post-operative period after third molar surgery, distinguishing among patients’ factors (age, sex, ethnicity, presence of systemic diseases, smoking, contraceptive therapy and oral hygiene), tooth-related factors (M3M inclination and angulation, depth of impaction, relationship to major anatomical structures, density of surrounding bone, occurrence of concomitant infective-inflammatory processes or neoplastic pathology), and operative factors (drug administration, type and extent of incision, necessity of bone removal, necessity of odontotomy, surgeon experience, and surgery time) [1, 2]. 

Different therapeutic protocols were implemented and analyzed to minimize postoperative sequelae, including nonsteroidal anti-inflammatory drugs (NSAIDs), laser treatment, steroids, ultrasound, platelet-rich fibrin (PRF) [3, 4]. To date, as reported by the most recent systematic reviews and meta-analyses, it’s hard to summarize the results to achieve solid conclusions because different methods of evaluation were used in the primary researches, mainly for facial edema assessment [5, 6]. Numerous methods of assessing and measuring postoperative swelling have been described in the literature, including bi-dimensional (2D) and three-dimensional (3D) methods (visual scoring or clinical observation, linear measuring devices, stereophotogrammetry, conventional computed axial tomography (CT), facial plethysmography, surface radiography, and surface laser scanners) [7–9]. Most of these techniques are operator-dependent without reproducible methodology. On the contrary, CBCT scans are more accurate, but with the disadvantage of exposing the patient to ionizing radiation. 3D surface meshes obtained by facial scanners are non-invasive techniques, but expensive, and often associated with private 3D reconstruction software. For this reason, the qualitative and quantitative evaluation of post-operative swelling continues to remain an important challenge to investigate the clinical, surgical, and therapeutic aspects of postoperative edema in the scientific research [10, 11]. Moreover, non-invasive extraoral and intraoral 3D scanning techniques, together with 3D printing procedures with biocompatible materials, are becoming increasingly popular in the medical field not only in diagnostic procedures, but also in therapeutic ones, through the design of customized devices, and in monitoring programs [12]. Although commercial companies offer facial scans with integrated tools for calculating mesh volumes, they are not open-source solutions, and they don’t allow to have a direct management of the images by clinicians. Therefore, the rationale of this study focused on the development of a methodological workflow for the 3D assessment of facial edema that is unambiguous, reliable, repeatable, operator-independent, and easily accessible, thus enabling direct management of the images by clinicians. The need for a new standardized and automated protocol stems from several limitations inherent in existing methods for assessing facial swelling post-surgery. Firstly, previous methods are based on subjective visual assessments with intra- and inter-operator variability, often focusing solely on linear measurements and neglecting the volumetric aspects of swelling, which are equally crucial for a comprehensive understanding of postoperative changes.

The aim of the study was to propose a standardized and automated protocol through open-source medical software for analyzing postoperative swelling after lower third molar surgery, enabling objective three-dimensional results to determine the clinical conditions immediately and one week after surgery.

## Methods

The study was designed as a prospective single center pilot study. The medical protocol and ethics followed the Declaration of Helsinki. Ethical committee approved the study (n. 465/2020). The inclusion criteria were the following: (1) age between 18 and 32 years; (2) no difference in gender or ethnicity; (3) group 1 of the American Society of 32 Anesthesiologists (ASA); (4) presence of prophylactic, strategic, or therapeutic indications for the removal of one (or both) M3M; (5) patients undergoing Cone Beam Computed Tomography (CBCT) scans. Patients with history of systemic diseases, mandibular trauma, current or previous therapy with bisphosphonates or radiation therapy, psychiatric disorders, cytopenic patients, or incomplete radiological exams were excluded.

After collecting anamnestic data and performing clinical evaluation, DICOM files were analyzed independently by two investigators (SB and MS) and each M3M was classified according to Juodzbalys & Daugela’s score of difficulty [13, 14]. Surgical procedures were conducted by the same expert maxillofacial surgeon (AG). If the patient required bilateral extractions, M3Ms were removed in two separate appointments a month apart. A single dose of antibiotic prophylaxis was administered 1 h before surgery: 2 g of amoxicillin or 600 mg of clindamycin in case of allergy. Immediately before surgery, the patient rinsed with a 0.20% chlorhexidine gluconate solution for one minute. The surgery involved performing a mucoperiosteal flap with osteotomy to dislocate and remove the tooth in the least traumatic way possible, followed by suturing the wound to facilitate healing. Post-operative instructions included pain relief therapy with 1 g of paracetamol per day for three days and meticulous oral hygiene procedures with 0.2% chlorhexidine.

**Fig. 1:**
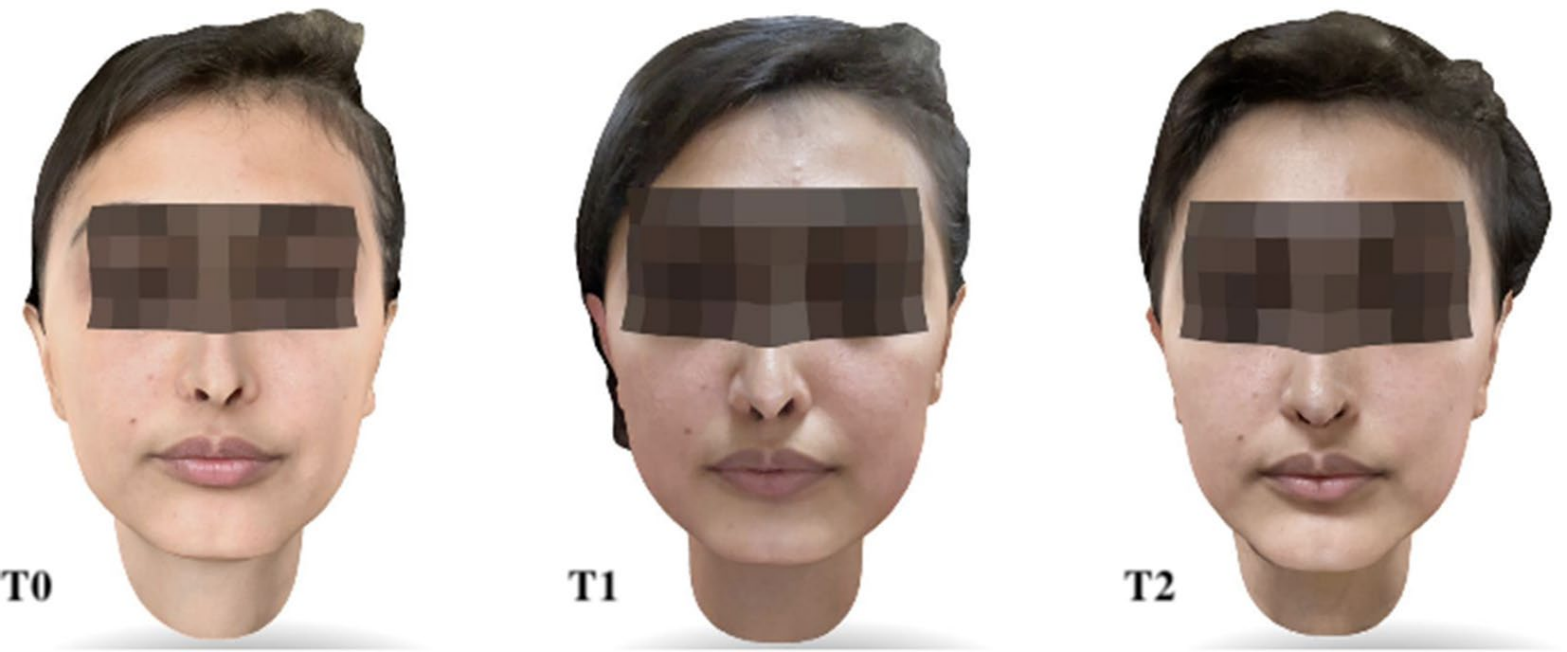
Facial scans were acquired using the 3D Bellus App at three different time points: before surgery (T0), three days after surgery (T1), and seven days after surgery (T2)

**Fig. 2:**
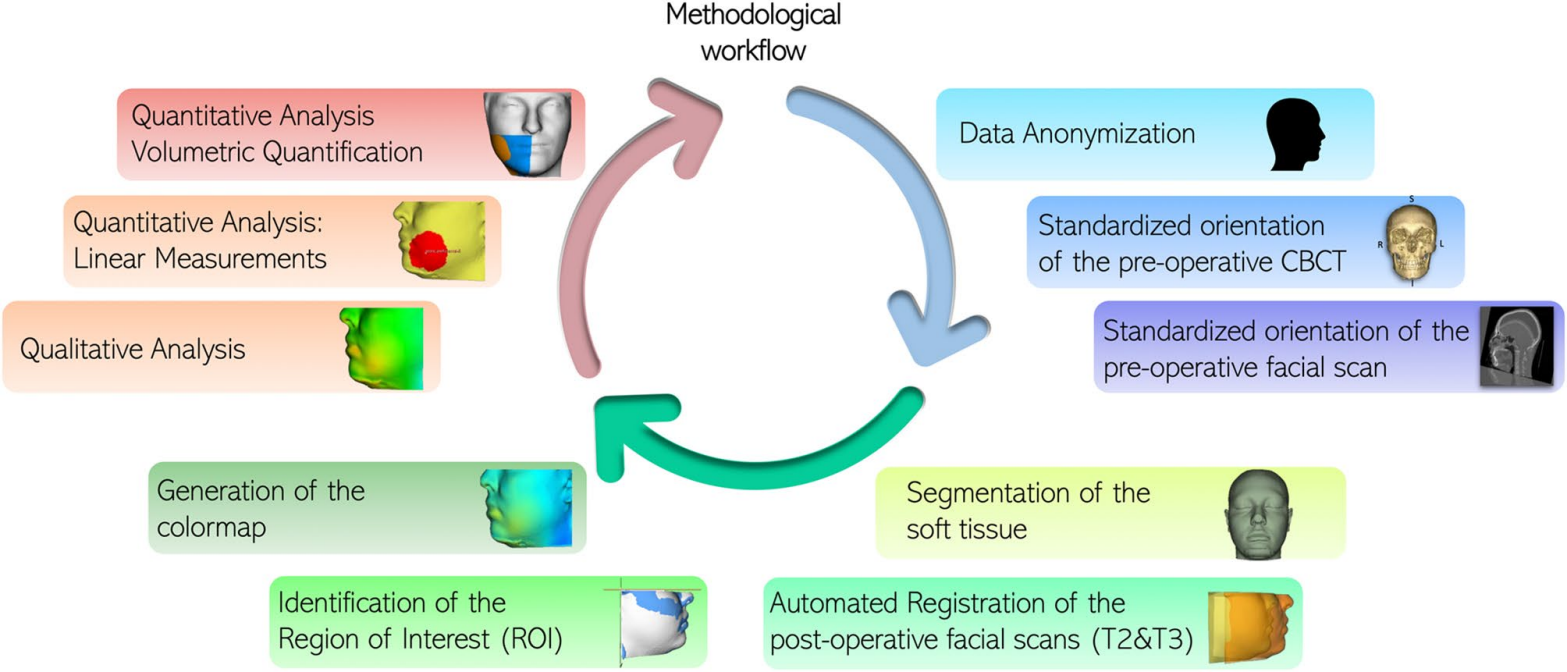
Automated pipeline for three-dimensional analysis of post-operative facial swelling

Study variables were registered before surgery (T0), three days (T1) and seven days (T2) after surgery. Facial swelling was considered the primary outcome variable. Using the scientifically validated 3D Bellus App (Bellus3D, versione 2.5.2, Bellus3D, Inc. Campbell, CA, USA) installed on an iPhone 12 Pro Max (Apple Store, Cupertino, CA, USA), three facial scans were performed, respectively at T0, T1 and T2 (Fig. 1) [15]. The scan was acquired by asking the patient to hold the jaw in a maximum intercuspation relationship, using the natural head position as a clinically reproducible method. The patient was asked to keep the facial muscles as relaxed as possible [16–19]. During the scanning phase, the device was fixed on a dedicated stand, while the patient followed the application’s voice instructions to rotate their head to the right, left, upward, and downward. To ensure consistent lighting conditions for each scan, a standardized environment was maintained for all patients, using only artificial lighting. Three-dimensional analysis was conducted using the open-source software 3D Slicer with automated tools able to reproduce the following 3D imaging workflow: (1) data anonymization; (2) standardized orientation of the pre-operative CBCT; (3) standardized orientation of the pre-operative facial scan; (4) segmentation of the soft tissue; (5) automated Registration of the post-operative facial scans (T2&T3); (6) identification of the Region of Interest (ROI); (7) generation of the colormap; (8) qualitative analysis; (9) quantitative analysis: linear measurements; (10) quantitative analysis: volumetric quantification (Fig. 2). Each facial scans set were imported as STL files and digital face models (visualization toolkit, vtk files) were generated. The automated surface registration algorithm enables the superimposition of two different surfaces in 3D space, automatically minimizing the distance between them. An automated surface registration of the T0 facial scan was performed on the 3D soft-tissue model segmented from the already oriented CBCT (standardized orientation according to the Frankfurt plane and the midsagittal plane) [20, 21]. For the comparisons, T1 and T2 facial scans were then registered on oriented T0 using the same procedure [11]. The automated tool Model-to-Model-Distance allowed the superimposition of the facial models in pairs as follow: T0-T1, to evaluate facial swelling three days after surgery; T1-T2, to evaluate any changes in facial edema between three and seven days after surgery; and T0-T2, to evaluate swelling occurrence one week after surgery. Qualitative analysis was performed after delimiting the region of interest identified by zygomatic arch superiorly, submandibular fossa inferiorly, preauricular region posteriorly, and the facial midline anteriorly. The tool ShapePopulationViewer created a colormap for each superimposed pair, aiming to highlight the specific localization of facial edema, and to compare differential swelling at different time-points, as shown by the colorbar. A visualization of the post-surgical changes can be automatically generated, focusing on the direction of movement too (vectors). The quantification of the soft tissues swelling was automatically achieved by calculating linear measurements (expressed in millimeters, mm) of the surface differences between the three paired models. Then, the plug-in Mesh Statistic was able to estimate a mean differential millimetric value.

### Automated volumetric assessment

The new extension “Mesh volume comparison” (https://github.com/pzaffino/SlicerMeshVolumeComparison) is already available in 3D Slicer and a unique package is provided to compact all the described tools of the workflow (already available at https://github.com/Micheladestito/SlicerSwellingEvaluation). It was used to quantify the volume difference between pair of models. In addition to the comparison task, it allows also to automatically close the open meshes given as input. For the specific edema assessment, the tool was able to record independently the single volume of each model or the difference between the three paired models. The computed volumetric output was provided in cubic millimeters, mm [3].

Secondary outcome variables included clinical assessments of: (1) pain, recorded using the Visual Analog Scale (VAS), from 0 (no pain) to 10 (worse pain) at the three timings; (2) trismus, measured as the distance in millimeters between the upper and lower incisal margins during the maximum mouth opening with a calibrated ruler; (3) surgery time, calculated by using a dedicated chronometer from the moment of incision to the beginning of the suture procedure.

Statistical analysis was performed using the software R (R version 4.3.1; http://www.r-project.org). Data were collected in a dedicated Excel file. A pilot study was conducted to determine the sample size needed for comparing the paired means. A total of 51 cases would be required, considering the following parameters: the difference in means (µ1-µ2 = 650.03), the standard deviation (SD = 1839.9), the significance level (α = 0.05), and the statistical power (β = 0.8). Descriptive statistics was reported, recording continuous variables as mean and standard deviation, while categorical variables as frequencies and percentages. The Kolmogorov-Smirnov and Shapiro-Wilk tests were used to determine the normal distribution of each variable in order to choose the more appropriate test for bivariate statistics. To evaluate the longitudinal changes for continuous variables, including the primary outcome variable (linear and volumetric differences) and secondary outcome variables (buccal opening, pain), the paired Student t-test was employed for normally distributed variables; alternatively, non-parametric tests were utilized. For the longitudinal changes in the categorical outcome variable (bleeding), a chi-square test was performed. Analysis of variance was employed to assess the primary and secondary outcome variables in relation to the primary predictor variable (score of difficulty). Additionally, a linear regression model was generated to correlate the primary outcome variables (linear and volumetric differences) with the secondary study variables (buccal opening, pain, bleeding, surgery time). Level of significance was established, setting α = 0.05.

## Results

**Fig. 3:**
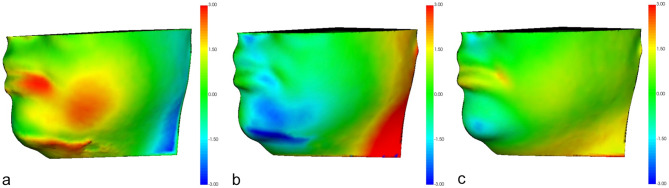
Qualitative analysis of a representative patient highlighting the differences between the pre-operative and post-operative facial scans. Semi-transparent overlays of 3D models and corresponding color-coded maps are displayed using a colorbar to show excess displacements (red) and deficits (blue). **a**) Lateral view of the facial swelling between T0 and T1. **b**) Lateral view of the facial swelling between T1 and T2. **c**) Lateral view of the facial swelling between T0 and T2

Demographic data are reported in Table 1. Qualitative analyses are reported in Fig. 3, which shows semi-transparent overlays, color-coded maps, and vector maps of facial scans at different timings (T0-T1, T1-T2, and T0-T2). The most significant discrepancy was recorded when comparing facial swelling at T1 with the initial condition (T0).

**Table 1:** Descriptive statistics

Study sample	
Patients (%)	35 (100)
Sex	
Female (%)	43 (79.6)
Age (years)	24.4 ± 5.8
Ethnicity	
Caucasian	34 (97.1)
Latin Americans	1 (2.9)
Smokers (%)	4 (7.4)
Third molars (%)	54 (100)
3.8	24 (44.4)
4.8	30 (55.6)
Roots apexification (%)	
G	4 (7.4)
H	50 (92.6)
Score of difficulty (%)	
0 – low	5 (9.3)
1 – mild	35 (64.8)
2 – moderate	11 (20.4)
3 – severe	3 (5.6)

The longitudinal quantitative analysis of the outcome variables showed a worsening of clinical conditions (facial swelling, pain, and trismus) immediately after surgery, followed by improvement one week after surgery in all patients (Tables 2 and 3).

**Table 2:** Longitudinal modifications of the primary outcome variable

Facial swelling	T0-T1	T1-T2	T0-T2	*p*-value*
Linear difference (mm)	1.6 ± 1.2	-0.8 ± 0.9	0.5 ± 0.6	**< 0.001**
Volumetric difference (mm^3^)	5649.2 ± 6977.6	-3813.5 ± 8309.6	1833.4 ± 6836.5	**0.00001**

*Comparison between T0-T1 and T1-T2

**Table 3:** Longitudinal modifications of the secondary outcome variables

Secondary outcome variables	T0	T1	T2	*p*-value
Buccal opening (mm)	43 ± 6.2	31.6 ± 10.4	36.6 ± 8.8	< 0.001*
				**< 0.001** ^#^
				**< 0.001**°
Pain	1.8 ± 2.8	4.9 ± 2.7	2.1 ± 2.3	< 0.001*
				0.4^#^
				**< 0.001**°
Bleeding (n)	3 (54)	0 (1)	0 (1)	> 0.05
		1 (52)	1 (46)	
		2 (1)	2 (7)	

*: Comparison between T0 and T1; ^#^: Comparison between T1 and T2; °: Comparison between T0 and T2; n = number of third molars

### Facial swelling

**Fig. 4:**
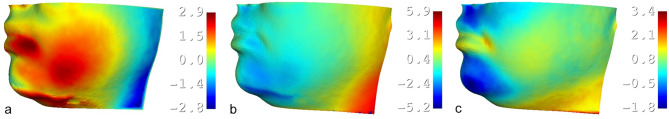
Quantitative analysis of a representative patient emphasizing the linear differences between the pre-operative and post-operative facial scans. The corresponding color-coded maps are displayed using a colorbar to show excess displacements (red) and deficits (blue). **a**) Linear measurement of the facial swelling between T0 and T1. **b**) Linear measurement of the facial swelling between T1 and T2. c) Linear measurement of the facial swelling between T0 and T2

**Fig. 5:**
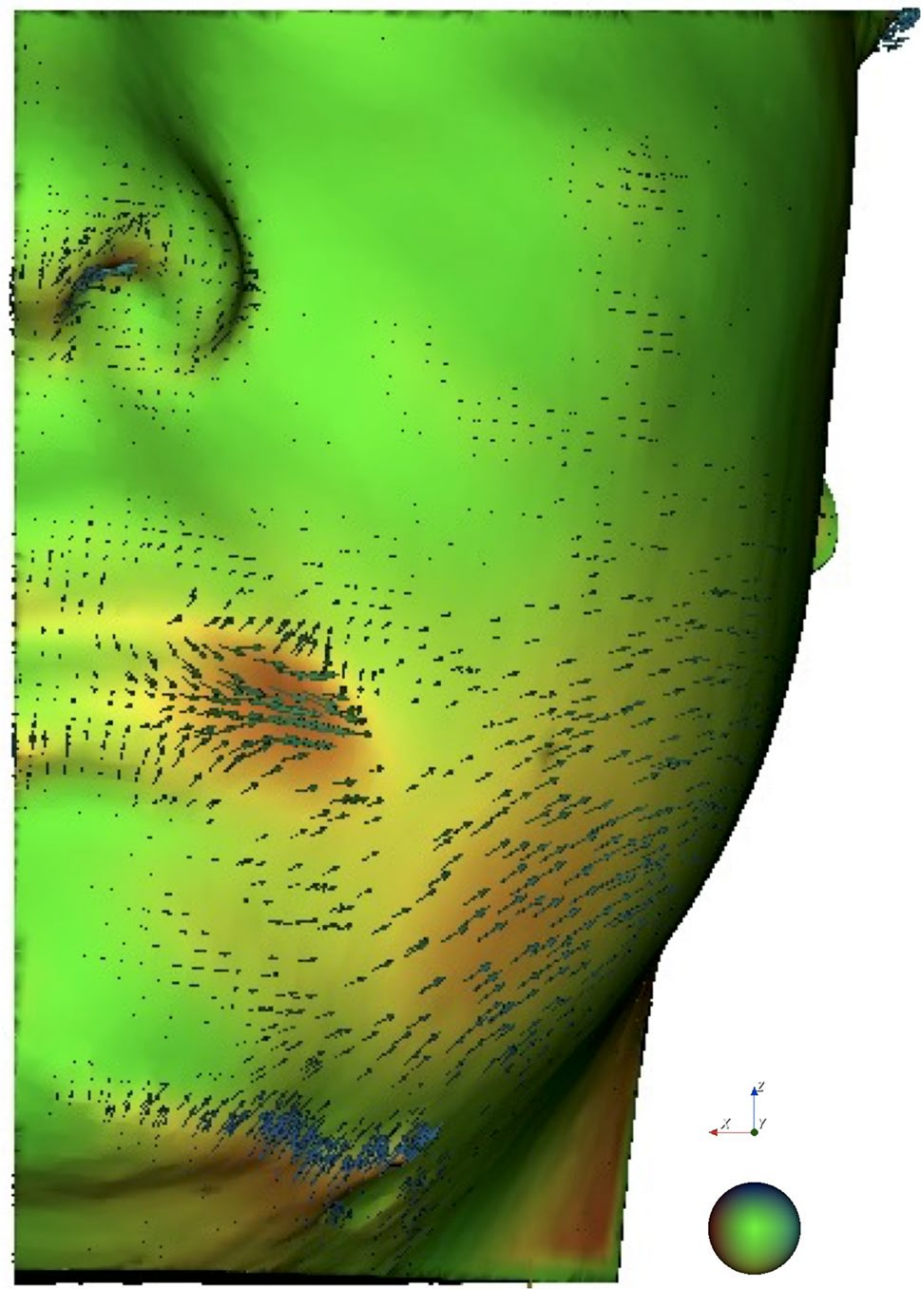
Visualization of the post-surgical changes using a 3D automated vectorial analysis highlighting the direction of displacements between pre-operative and post-operative facial scans

**Fig. 6:**
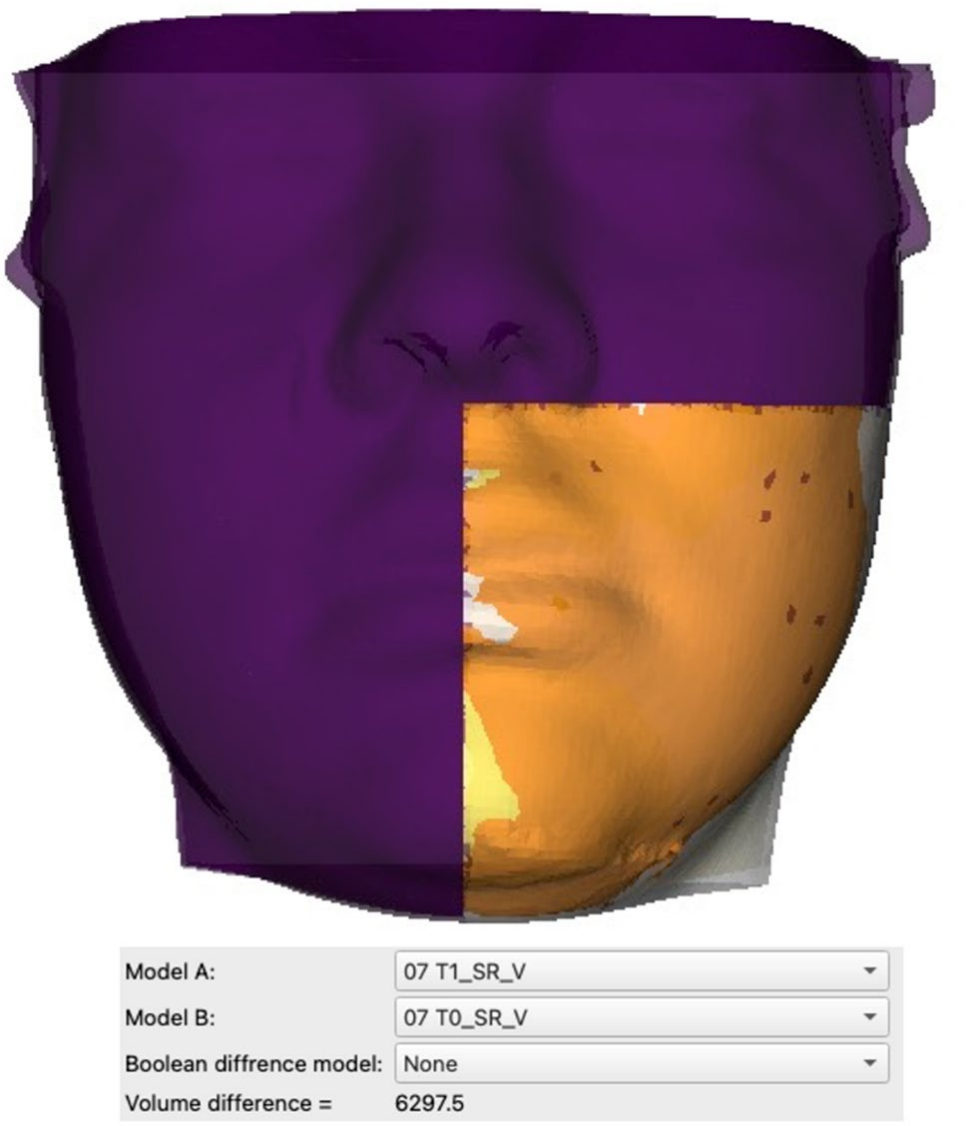
Quantitative automated analysis of the volumetric differences in the region of interest between the pre-operative and post-operative facial scans using the new extension “Mesh volume comparison” in 3D Slicer

Table 4 reports the analysis of variance of the primary outcome variable in relation to the primary predictor variable. No statistically significant differences were found for either linear difference or volumetric difference (*p* > 0.05) (Figs. 4 and 5-6). Pearson’s analysis showed a positive correlation between the primary predictor variable (linear difference T0-T1, linear difference T1-T2, volume difference T0-T1, volume difference T1-T2) and surgery time (*p* < 0.05). Linear regression analysis is reported in Table 5. There was a direct correlation between linear difference T0-T1 and linear difference T0-T2, as well as volume difference T0-T1, while an inverse correlation was observed between linear difference T0-T1 and linear difference T1-T2, as well as volume difference T0-T2 (*p* < 0.05). Additionally, there was a direct correlation between linear difference T0-T2 and linear difference T0-T1, surgery time, and volume difference T0-T2, whereas an inverse correlation was found between linear difference T0-T2 and volume difference T0-T1 and T1-T2 (*p* < 0.05). Moreover, a direct correlation was observed between linear difference T1-T2 and buccal opening at T0, as well as volume difference T0-T2, while an inverse correlation was noted between linear difference T1-T2 and buccal opening at T2, linear difference T0-T1, pain at T1, and volume difference T0-T1 and T1-T2 (*p* < 0.05). Furthermore, there was a direct correlation between volume difference T0-T1 and volume difference T0-T2, while an inverse correlation was found between volume difference T1-T2 and volume difference T1-T2 (*p* < 0.05). Additionally, a direct correlation was observed between volume difference T1-T2 and volume difference T0-T2, while an inverse correlation was noted between volume difference T1-T2 and volume difference T0-T1 (*p* < 0.05). Lastly, there was a direct correlation between volume difference T0-T2 and volume difference T0-T1 and T1-T2 (*p* < 0.05).

**Table 4:** Analysis of variance of the primary outcome variable (swelling) in relation to the primary predictor variable

Study outcome	Score of difficulty				*p*-value
	0 – low	1 – mild	2 – moderate	3 – severe	
Linear difference T0-T1 (mm)	0.63 [0.10, 1.69]	1.40 [-0.21, 3.98]	1.90 [0.60, 6.30]	1.76 [0.33, 2.08]	0.2
Linear difference T0-T2 (mm)	0.30 [-0.22, 2.34]	0.32 [-0.59, 1.74]	0.42 [-0.12, 1.22]	0.46 [-0.34, 1.08]	0.9
Linear difference T1-T2 (mm)	-0.09 (0.68)	-0.86 (0.95)	-0.76 (0.97)	-0.98 (0.59)	0.4
Volume difference T0-T1 (mm^3^)	4366.7 [-2264.3, 14396.9]	4171.4 [-4681.6, 21980.0]	5777.6 [1390.1, 38579.5]	5378.3 [409.8, 6359.6]	0.8
Volume difference T0-T2 (mm^3^)	836.0 [213.1, 33447.3]	806.7 [-23319.2, 14520.0]	-60690.4 [-38052.1, 287.9]	-3686.7 [-4571.6, -1263.1]	0.9
Volume difference T1-T2 (mm^3^)	2744.3 [-5211.8, 19050.4]	-3187.0 [-27690.4, 13249.6]	1701.9 [-7514.4, 4023.5]	806.7 [-3276.9, -5096.5]	0.3

**Table 5:** Linear regression model of the primary outcome variable (facial swelling)

Linear difference T0-T1		
Study variable	Estimate	p-value
Intercept	0.36	**0.01**
Linear difference T0-T2	0.88	**< 0.001**
Linear difference T1-T2	-0.33	**0.004**
Volume difference T0-T2	-0.00004	**0.04**
Volume difference T0-T1	0.001	**< 0.001**
Linear difference T0-T2		
Study variable	Estimate	p-value
Intercept	-0.17	0.2
Linear difference T0-T1	0.24	**0.001**
Surgery time	0.02	**0.003**
Volume difference T0-T2	0.01	**0.03**
Volume difference T1-T2	-0.01	**0.03**
Volume difference T0-T1	-0.01	**0.03**
Linear difference T1-T2		
Intercept	-1.95	**0.01**
Buccal opening T0	0.09	**0.0001**
Buccal opening T2	-0.04	**0.005**
Linear difference T0-T1	-0.44	**0.0005**
Pain T1	-0.1	**0.02**
Volume difference T0-T2	0.01	**0.04**
Volume difference T1-T2	-0.01	**0.04**
Volume difference T0-T1	-0.01	**0.04**
Volume difference T0-T1		
Intercept	0.04	**< 0.001**
Volume difference T0-T2	1.0	**< 0.001**
Volume difference T1-T2	-1.0	**< 0.001**
Volume difference T1-T2		
Intercept	0.02	**< 0.001**
Volume difference T0-T2	1.0	**< 0.001**
Volume difference T0-T1	-0.99	**< 0.001**
Volume difference T0-T2		
Intercept	-0.02	**< 0.001**
Volume difference T1-T2	0.99	**< 0.001**
Volume difference T0-T1	0.99	**< 0.001**

### Secondary outcome variables

Table 6 shows the analysis of variance of the secondary outcome variables based on the difficulty score. No statistically significant differences were found (*p* > 0.05).

**Table 6:** Analysis of variance of the secondary outcome variables in relation to the primary predictor variable

Study outcome	Group	Score of difficulty				*p*-value
		0 – low	1 – mild	2 – moderate	3 – severe	
Surgery time (min)		9.9 (4.9)	21.5 (10.9)	21.3 (6.3)	18.4 (5.9)	0.09
Buccal opening T0 (mm)		45.8 (9.7)	43 (5.6)	42.8 (6.2)	40 (8.7)	0.6
Buccal opening T1 (mm)		38 (11.1)	32.1 (9.7)	28.4 (11.6)	27.2 (11.1)	0.3
Buccal opening T2 (mm)		40.1 (8.1)	37.6 (7.8)	33.2 (9.8)	31.0 (14.8)	0.2
Pain T0		0.0 [0.00, 1.00]	0.0 [0.00, 8.00]	0.0 [0.00, 6.00]	0.00 [0.00, 0.00]	0.3
Pain T1		4.00 [2.00, 7.00]	5.00 [0.00, 9.00]	6.00 [0.00, 9.00]	4.00 [0.00, 7.00]	0.6
Pain T2		2.00 [0.00, 4.00]	2.00 [0.00, 9.00]	2.00 [0.00, 8.00]	3.00 [0.00, 5.00]	0.7
Bleeding T0 n (%)	3	5 (100)	35 (100)	11 (100)	3 (100)	> 0.05
Bleeding	0	0 (0)	1 (2.9)	0 (0)	0 (0)	
T1	1	5 (100)	33 (94.3)	11 (100)	3 (100)	
n (%)	2	0 (0)	1 (2.9)	0 (0)	0 (0)	
Bleeding	0	0 (0)	1 (2.9)	0 (0)	0 (0)	
T2	1	5 (100)	29 (82.9)	10 (90.9)	2 (66.7)	
n (%)	2	0 (0)	5 (14.3)	1 (9.1)	1 (33.3)	

n = number of third molars

## Discussion

In the last decades, three-dimensional diagnostic tools and a better quality of the soft tissues’ evaluation became fundamental for the consistent improvement of the available software of image analysis [11, 22, 23]. To date, objective 3D assessments of the post-operative swelling after surgical procedures in the head and neck region were accessible only in combination with specific facial scanners [23, 24]. For this reason, the purpose of this study was to introduce an innovative automated method for the measurement of the post-surgical swelling provided by an open-source medical software.

The applicability of this process was clinically reported including candidates to lower third molar surgery, one of the most common causes of inflammation in oral surgery. Soft tissues’ detection was objectively recorded, collecting facial scans at different time-points during the first post-operative week. Facial scanner is not an invasive tool, able to provide accurate boundaries of the face, and considered as a safe and reliable procedure [24, 25]. The automated comparative analysis in the software 3D Slicer allowed to better identify the post-operative edema, considering the cheek, the submandibular area and the preauricular area as regions of interest [19, 25]. For the qualitative analysis, a visualization of the post-surgical changes can be automatically generated, focusing on the direction of movement too. The quantitative analysis can provide detailed data on linear and volumetric measurements at different time-points, also including the opportunity to correlate these results with the clinical outcomes. The entire pipeline is not dependent on the fine-tuning of some technical patient-specific parameter, minimizing the risk of jeopardize the investigation. In addition to the comparison task, this extension allows also to close open meshes, enabling a more reliable investigation of no-closed models that is a common scenario in such clinical studies. Furthermore, quantitative differences were computed by using open-source software, enabling a cost-free reproducibility of the workflow. Considering the valid outcome and the good quality assessment, the algorithm is already available for clinical and research purpose.

This longitudinal analysis demonstrated a deterioration of clinical conditions (facial swelling, pain, and trismus) immediately after surgery, followed by improvement one week after surgery in all patients. Based on the difficulty score, no statistically significant differences were found in terms of facial swelling, pain, and trismus. However, third molars classified as moderate to severe exhibited higher post-operative inflammatory signs and symptoms. These findings should be interpreted in light of the varying number of third molars in each group. The sample used in this study was sourced exclusively from the university clinic, where simple M3Ms are less common due to their prevalence in private practices. Academic clinics primarily handle mild and moderate M3M cases, resulting in a higher representation in this study. Conversely, the severe third molars group comprises fewer cases, as they may not meet removal criteria, making post-surgical evaluation unfeasible. To further support the influence of different difficulty levels on post-operative outcomes, Pearson’s analysis revealed a positive correlation between facial swelling and surgery time, both in terms of linear and volumetric differences. There was a direct proportionality between linear and volume differences, whereby an initial increase immediately after surgery was associated with a subsequent increase one week after surgery.

As reported in literature, facial swelling has been widely assessed as a significant indicator of the postsurgical inflammatory reaction and different methods was used for its quantification. Particularly referred to lower third molar surgery, no consistency nor standardized measurement method were recorded in swelling evaluation [26]. Quantifying swelling after third molar surgery is a challenging variable to measure accurately due to the uneven surface being measured. Papazov and Burschka have emphasized the need for an automatic and robust registration procedure, which enhances the reliability of facial swelling analysis results [27]. Most studies performed linear measurements of the post-operative alteration of the soft tissue profile, considering horizontal, vertical, and oblique distance between specific anatomic landmarks in the hemiface ipsilateral to the surgery [26, 28–35]. Calipers, face bows, tape, and flexible rulers were the available tools for this calculation, but limited data could be achieved [19, 35]. In the past, to detect facial edema two-dimensional methods were also applied by the analysis of frontal radiographs with a barium-containing suspension painted on the skin and by the comparison of facial photographs [36, 37]. However, in the first case, the radiation dose for the patient could interfere with the purpose, while the second option could be negatively influenced by the acquisition’s procedures [25]. More recently, some authors also described the use of stereophotographic methods with 3D laser-scanning devices to quantify post-operative facial swelling with volumetric measurements [38–41]. The availability of 3D scanning technology is becoming increasingly widespread due to the development of mobile applications employing TrueDepth sensors in smartphones. Several studies have positively explored their potential clinical use for assessments that are within specific accuracy ranges [15]. Although for some regions of the face, mobile scanning technology suffers from restricted levels of accuracy, its use is becoming increasingly popular due to its ease-of-use, convenience and ease-of-access features, which are expected to be associated with a gradual optimization of scan quality [17]. The region of interest after lower third molar surgery was manually identified by locating four surface landmarks. Although this procedure could be more accurate and reproducible compared to the previous techniques, its success is still limited by the complex equipment and high cost of the software [33, 40]. The most recent systematic reviews and meta-analyses confirmed this extreme variability in post-surgical swelling assessment, recommending the introduction of a 3D standardized protocols in the future clinical studies [33, 34, 40]. Nogueira, in his meta-analysis, recommends the implementation of standardized, precise, and controlled measurement protocols to enable reliable comparisons between different investigations related to the outcomes of third molar surgery. Swelling represents a volumetric change, and the most accurate assessment can only be achieved with three-dimensional measurements. More consistent initial measurements and more precise data processing were considered fundamental pillars for the comparison of edema outcomes intra- and inter-studies [34]. 

The major strength of this study is the automated methodological protocol for post-operative swelling analysis, able to provide objective three-dimensional results that indicate swelling, pain, and trismus are correlated with surgery time and commonly observed three days after surgery with significant improvement seven days post-surgery. The application of this automated methodological protocol for post-operative swelling analysis overcomes some critical issues related to validity, costs, and repeatability of these assessments since 3D Slicer is an open-source medical software. Furthermore, using facial scans as input allowed to detect the primary outcome at different and close timings without any invasiveness for the patient. The pre- and post-surgical comparisons in this study were both accurate and reliable. This was achieved through analysis in three-dimensional space planes, utilizing established mathematical models for precision. All comparisons were conducted using automated superimposition schemes, ensuring operator-independence. However, the study is not free of limitations. First, only outcomes related to lower third molar surgery were assessed, but for the simplicity in acquiring facial scans, no restrictions could be found in extending this protocol to other treatment. Although a sample size calculation was performed on the data of a pilot trial, it was designed a single-centre study with a relatively small sample size, which may represent a further limitation. An open-source, standardized, automated and easily accessible analysis workflow, allows to be simply extended studies on the assessment of facial edema after third-molar surgery to other centers. As a powerful 3D medical software, a learning curve could be requested to become familiar with the procedure, but online tutorials already exist and may be helpful for this purpose. Finally, a standardized orientation of the T0 facial scan was performed before each comparison, using the soft tissues’ segmentation of the oriented presurgical CBCT as reference. Although orientation doesn’t interfere with the mathematical results, it is still the first step of imaging analysis, allowing to relocate the skull in a proper position in the three spatial axes. The future goal is to develop a dedicated tool for automated orientation of facial scans, utilizing artificial intelligence and machine learning approaches. This would also facilitate the development of future studies comparing the new protocol with existing methods of assessing facial edema, as well as subsequent comprehensive reviews and accurate meta-analyses on heterogeneous and objective data. The availability of this innovative method of analysis promotes new trials to monitor facial edema by comparing different therapies, to provide an objective answer on the most appropriate treatment approach to be taken. Through a standardized and operator-independent workflow, investigating the mechanism of facial swelling after specific surgeries can be beneficial to clinicians, who will thus be able to understand the post-operative conditions and implement appropriate medical choices aimed at not compromising the patient’s quality of life.

## Conclusion

In conclusion, this study employed a comprehensive digital workflow of image analysis, which allowed for accurate quantification of post-operative facial changes. Through the implementation of a new tool, volumetric measurements were achieved for the first time, enhancing the precision and depth of these findings. The utilization of this advanced technology provided unprecedented insights into the dimensions of facial changes, surpassing the limitations of traditional linear measurements. Clinically, the study results indicate an initial increase in swelling, trismus, and pain three days after surgery, followed by improvement at the one-week mark. The post-operative inflammatory outcomes show a direct increase with longer surgery times, both immediately and one week after surgical treatment. This novel approach holds great promise for future studies in the field, offering researchers and practitioners a powerful tool for objective and reliable evaluation of facial changes.

## Data Availability

The datasets used and/or analyzed during the current study are available from the corresponding author on reasonable request.

## Abbreviations

M3M: mandibular third molars
NSAID: nonsteroidal anti-inflammatory drugs
PRF: platelet-rich fibrin
2D: bi-dimensional
3D: three-dimensional
CT: computed axial tomography
CBCT: cone beam computed tomography
ASA: American Society of 32 Anesthesiologists
T0: before surgery
T1: three days after surgery
T2: seven days after surgery
VAS: Visual Analog Scale
